# Optimization of bioprocesses with Brewers’ spent grain and *Cellulomonas uda*

**DOI:** 10.1002/elsc.202100053

**Published:** 2021-08-27

**Authors:** Alexander Akermann, Jens Weiermüller, Jonas Nicolai Chodorski, Malte Jakob Nestriepke, Maria Teresa Baclig, Roland Ulber

**Affiliations:** ^1^ TU Kaiserslautern Department of Mechanical and Process Engineering Kaiserslautern Germany

**Keywords:** bioprocess optimization, Brewers’ spent grain, *Cellulomonas uda*, solid‐state fermentation

## Abstract

Brewers’ spent grain (BSG) is a low‐value by‐product of the brewing process, which is produced in large quantities every year. In this study, the lignocellulosic feedstock (solid BSG) was used to optimize fermentations with *Cellulomonas uda*. Under aerobic conditions, maximum cellulase activities of 0.98 nkat∙mL^−1^, maximum xylanase activities of 5.00 nkat∙mL^−1^ and cell yields of 0.22 g_Cells_∙g_BSG_^−1^ were achieved. Under anaerobic conditions, enzyme activities and cell yields were lower, but valuable liquid products (organic acids, ethanol) were produced with a yield of 0.41 g_Prod_∙g_BSG_^−1^. The growth phase of the organisms was monitored by measuring extracellular concentrations of two fluorophores pyridoxin (aerobic) and tryptophan (anaerobic) and by cell count. By combining reductive with anaerobic conditions, the ratio of ethanol to acetate was increased from 1.08 to 1.59 mol_EtOH_∙mol_Ac_^−1^. This ratio was further improved to 9.2 mol_EtOH_∙mol_Ac_^−1^ by lowering the pH from 7.4 to 5.0 without decreasing the final ethanol concentration. A fermentation in a bioreactor with 15 w% BSG instead of 5 w% BSG quadrupled the acetate concentration, whilst ethanol was removed by gas stripping. This study provides various ideas for optimizing and monitoring fermentations with solid substrates, which can support feasibility and incorporation into holistic biorefining approaches in the future.

**Abbreviations**
BLAST: Basic Local Alignment Search Tool
BSG: Brewers’ spent grain
cys‐HCl: cysteine hydrochloride
DSMZ: German Collection of Microorganisms and Cell Cultures
E.C.: enzyme commission
HHV: higher heating value
KEGG: Kyoto Encyclopedia of Genes and Genomes database
M: million
NREL: National Renewable Energy Laboratory
OS: organosolv
USD: US dollar

## 1 INTRODUCTION

Lignocellulosic feedstock, e.g. Brewers’ spent grain (BSG), has been the subject of research for a long time. However, the purpose of the research changed over the years. As a result of the 1973 oil crisis, lignocellulosic biomass came into focus as an energy source and feedstock for chemical synthesis, because of the sudden shortage of fossil fuels [1]. In the following years and decades, it was assumed that oil prices would continue to rise steadily—not necessarily because of political and military conflicts, but because of the natural shortage of crude oil. In reality, however, the prices hardly changed until 2000 [2]. This misjudgement led to various proposals for the application of lignocellulosic feedstock, e.g. as fertilizer [3] but also an increased interest in producing bulk chemicals like ethanol from wooden biomass [4]. During this period, the basis for research on *Cellulomonadaceae* was established, especially in regards to the production of lignocellulose degrading enzymes and the physiological properties of this family [5, 6, 7, 8, 9, 10, 11, 12, 13, 14, 15, 16]. Nowadays, research on lignocellulosic biomass is not due to the pressure of the crude oil shortage, but is related to key issues and initiatives like global warming [17], greenhouse gas emissions [18], petroleum saving [19] and circular economy [20]. Until now, the applicability of *Cellulomonadaceae* for technical devices was demonstrated for the removal of heavy metals and radionuclides from wastewater [21], the production of single cell proteins [22], cellulases [23] as well as xylanases [24] and the generation of electricity in microbial fuel cells [25]. Increasing interest in this genus can also be seen from the large number of recently isolated new strains, such as: Cellulomonas* telluris* [26], *Cellulomonas timonesis* [27], *Cellulomonas endophytica* [28], *Cellulomonas aurantiaca* [29] and *Cellulomonas algicola* [30].

BSG is one of the most abundant lignocellulosic resources from an industrial process with a production capacity of 39 M tons∙yr^−1^ and a price of approximately 39 USD∙ton^−1^ [31, 32]. The potential of BSG was already demonstrated by direct solid‐state fermentations with *Rhizopus oligosporus* or *Bacillus subtilis *WX‐17 to increase the nutritional value [33, 34], or by processing with various organisms to produce enzymes like laccases, cellulases and lipases [35, 36]. However, the production of bulk chemicals like alcohols or organic acids from BSG is mainly described in two‐ or three‐stage processes, consisting of hydrothermal pretreatment, enzymatic saccharification and subsequent fermentation [37, 38, 39, 40]. An alternative is pointed out by co‐fermentation of a lignocellulose‐degrading organism with a heterotrophic production strain [41]. A summary of recently described processes for the usage of BSG and other lignocellulosic substrates (in terms of ethanol and acetate production) can be seen in Table 1. *Cellulomonas uda*, the organism applied in this study, is a non‐motile, gram‐positive, coryneform, facultative anaerobic bacterium, which has the ability to hydrolyze cellulose, hemicellulose, chitin and perform a mixed‐acid fermentation [6, 15, 42]. Drawing from these previous applications, a new application for the genus *Cellulomonas* is in the degradation of the lignocellulosic biomass and the production of valuable products in one consolidated bioprocess. The discovery of these consolidated fermentations with high solids loadings includes both advantages and disadvantages: An advantage is that the high solid (substrate) loadings lead to decreased water demand and potentially high volumetric productivities [43]. In addition, less energy and chemicals will be used, since costly hydrothermal and enzymatic pretreatments do not have to be carried out. On the other hand, a disadvantage is that product concentrations are often limited by poor degradability of the lignocellulosic feedstock, called biomass recalcitrance [44]. Furthermore, monitoring these processes is challenging due to the complexity and heterogeneity of the medium, sensor disruption, limitations in heat and nutrient transfer and because of the bioreactor design fulfilling all the requirements for the processes. In particular, the determination of cells by conventional (gravimetrical or optical) methods is difficult. Therefore, indirect methods, such as respiratory quotients, pressure change in closed systems or infrared spectroscopy, are mostly used [45, 46, 47].

**PRACTICAL APPLICATION**

As Brewers’ spent grain ist an abundant by‐product of the brewing process, this study makes a contribution to develop processes for its application in white biotechnology. In particular, this applies to monitoring and optimization strategies for consolidated bioprocesses with solid lignocellulosic substrates, which can also be applied to other organisms and substrates in the future. This is of special interest as biorefining approaches might be one solution in overcoming recent global problems.

**TABLE 1:** Summary of recently described processes for the production of ethanol and acetate from lignocellulosic feedstock in terms of process set‐up and final product concentrations

Substrate	Process set‐up	Final ethanol concentration /g∙L^−1^	Final acetate concentration /g∙L^−1^	Reference
Oil palm trunk	(a) Saccharification by fungal isolates	4.15	2.12	[48]
	(b) Fermentation by *Saccharomyces cerevisiae* and *Acetobacter aceti*			
Brewers’ spent grain	(a) Alkaline‐acid treatment	12.79	n.a.	[49]
	(b) Enzymatic saccharification			
	(c) Fermentation with *S. cerevisiae*			
Brewers’ spent grain	(a) Pretreatment: autohydrolysis	42.27	n.a.	[37]
	(b) Enzymatic saccharification and parallel fermentation by *S. cerevisiae*			
Brewers’ spent grain	(a) Phosphoric acid pretreatment	16	n.a.	[50]
	(b) Enzymatic saccharification			
	(c) Fermentation by *Escherichia coli*			
Brewers’ spent grain	(a) Saccharification by filamentous fungi, e.g. *Aspergillus oryzae*	37	n.a.	[41]
	(b) Fermentation by yeasts, e.g. *S. cerevisiae*			
Wheat straw	(a) Phosphoric acid plus hydrogen peroxide pretreatment (b) Enzymatic saccharification (c) Fermentation by *S. cerevisiae*	71.2	n.a.	[51]
Japanese cedar	(a) Pretreatment with acetone and hot‐compressed water	n.a.	0.8	[52]
	(b) Co‐fermentation of Clostridium* thermoaceticum* and *Clostridium thermocellum*			
Corn stover or wheat straw hydrolysate	(a) Wet explosion pretreatment (b) Enzymatic saccharification (c) Fermentation with Acetobacterium* woodii*	n.a.	7.64–7.83	[53]

Abbreviation: n.a., not available.

In this publication, fermentations with BSG are the subject of investigation using the organism *C. uda*. First, the effects of aerobic and anaerobic conditions on the process are investigated in suspension cultures. These experiments are also used to develop methods for process analytics including strategies for cell determination, the measurement of fluorophores, as well as the calculation of microbial enzymatic activity. Besides, a holistic feedstock description during the fermentations is provided. Furthermore, this study investigates how process technology and cultivation conditions affect the metabolism of the organisms, and as a result affect the product formation mainly in terms of ethanol and acetate. As a final step, the fermentation is transferred to a bioreactor and performed with higher solid contents (leading to a pulpy substrate). All results are discussed in terms of the central metabolism of *C. uda*, which is visualized for the first time based on bioinformatical alignments with well‐known *Cellulomonadaceae*.

## 2 MATERIALS AND METHODS

The values shown in this study (e.g. concentrations and yields) represent the mean value (± standard deviation) of two or three independent biological replicates. If this is not the case, it is indicated in the specific section.

### 2.1 Feedstock generation

BSG was produced as a side product of a *May bock* brewing process in a 100 L pilot plant. Directly after the lautering process, wet BSG was pressed four times by a tincture press (Hochdrucktinkturenpresse HP 2 H, Fischer Maschinenfabrik GmbH, Neuss, Germany) at *p* = 200 bar in order to remove all soluble components, mainly sugar [54, 55]. The solid residue was rehydrated after each pressing step with deionized water equalling the volume of the liquid fraction removed. Since the mentioned pressing step resulted in numerous advantages, dextrin‐ and sugar‐free BSG were also tested in this study in order to achieve a more holistic perspective on the utilization of BSG (biorefining concept) [54, 55]. Afterwards, the BSG residue was dried at 323 K for 48 h (UF110, Memmert GmbH & Co. KG, Schwabach, Germany) and ground to fine particles by a disc mill (Fidibus Classik, Komo GmbH & Co. KG, Hopfgarten, Germany). Finally, the ground residue was vacuumed and stored at 253 K until usage.

### 2.2 Strain and cryo conservation

*C. uda* DSM 20108 was obtained by the German Collection of Microorganisms and Cellcultures (DSMZ GmbH, Braunschweig, Germany). The cells were grown in aerobic suspension cultures, which consisted of 0.2 M MOPS buffer (pH = 7.4) and 5 w% of ground BSG. Aliquots of the fermentation broth were harvested after 4 days of fermentation and mixed with an equal volume of 80 vol% glycerol. The stock cultures were shock‐frozen with liquid nitrogen and stored at 193 K until further use (Ultra Low Temperature Freezer DW‐86L388J, Haier Biomedical, Qingdao, China).

### 2.3 Investigation of aerobic and anaerobic process conditions

Two independent biological duplicates of *C. uda* were grown in order to investigate the growth of the bacteria under the presence or absence of oxygen. For anaerobic conditions, the fermentations were performed in 250  mL flasks (Schikanenkolben GL 45 thread, DWK Life Sciences, Wertheim/Main, Deutschland), which were sealed with septa (Butyl stopper ‐ massive, Glasgeraetebau Ochs, Laborfachhandel e.K., Bovenden, Germany). Anaerobic conditions were achieved by fumigation with sterile nitrogen through a hollow needle for 2 h. Aerobic cultures were grown in 300 mL baffled Erlenmeyer flasks, which were closed with cotton plugs. In both cases, the fermentation medium (100 mL) consisted of 5 w% BSG and 5 g∙L^−1^ yeast extract in 0.2 M MOPS buffer at pH = 7.4. The inoculation occurred with a cryo culture, which was prepared as described in Section 2.2. The cultures were incubated at T = 303 K, N = 120 rpm and a lift of 25 mm in a shaking incubator (Ecotron, Infors AG, Bottmingen, Switzerland). The pH was adjusted manually every 24 h through the addition of (anaerobic) 5 M NaOH, according to an empirically determined Equation (1). All media components were autoclaved (V‐150, Systec GmbH, Linden, Germany), but BSG/yeast extract and buffer were autoclaved separately to prevent hydrothermal degradation of the feedstock.

(1) $$\;V_{NAOH}=\frac{pH_{target}-pH_{current}}{47.85}\;\cdot V_{Culture,\;current}$$

Product concentrations were determined by using high‐performance liquid chromatography (HPLC), as described in the supporting information.

### 2.4 Optimization of the process

Varying anaerobic fermentation conditions and supplements were evaluated in order to reach three goals: (I) investigate the metabolic response of *C. uda* to changing media conditions, (II) increase the maximum product concentration and (III) force the metabolism to produce mainly one product (here: ethanol) and accordingly decrease side product formation. The basic fermentation medium consisted of 5 w% BSG in anaerobic 0.2 M MOPS buffer. This basic medium was altered as stated below:

The initial pH of the fermentations was set to 5, 7.4 and 9, respectively. In some experiments one or more of the following components were added to the medium [g∙L^−1^]: yeast extract [5], cysteine hydrochloride (cys‐HCl) [1] and K_3_FeCN_6_ [0.329]. The concentrations of cys‐HCl and K_3_FeCN_6_ were adopted from literature [54, 56]. In addition, the effects of removing the pressing step, as described in chapter 2.1, were investigated; therefore soluble sugar and dextrin were present in the fermentation medium.

To facilitate the bacterial degradation of BSG, an organosolv (OS) treatment was performed for pressed BSG with a 50 vol% ethanol‐water mixture at T = 433 K for 10 min in a high‐pressure lab reactor (BR‐500, Berghof Products + Instruments GmbH, Eningen, Germany). The washed residue of this treatment was used for two fermentations at pH = 7.4, and pH = 7.4 with addition of 1 g∙L^−1^ cys‐HCl and 5 g∙L^−1^ yeast extract. In further fermentations, commercial cellulases (NS22192) and hemicellulases (NS22083), both from Novozymes A/S, Bagsvaerd, Denmark, were added with concentrations of 0.08 g_Enzyme_∙g_BSG_^−1^ as a single enzyme mixture or in combination.

All experiments were performed in independent biological duplicates, except the fermentations with OS‐treated BSG, which were performed as single experiments due to feedstock limitations.

Inoculation, incubation parameters, sterilization of media components and pH regulation were adopted from Section 2.3.

### 2.5 Growth conditions in a bioreactor

An aerobic preculture (100 mL) was grown for 5 days in a 300 mL baffled Erlenmeyer flask as described in Section 2.3 to reach a high biomass concentration as well as high cellulase and xylanase activities. Twenty‐five millilitres of the preculture was used to inoculate 350 mL of the main culture, which was grown in a bioreactor (Biostat Q Plus, Sartorius AG, Goettingen, Germany). This main culture consisted of 15 w% BSG, 15 g∙L^−1^ yeast extract and 1 g∙L^−1^ cys‐HCl in 0.2 M MOPS buffer at pH = 7.4. The increase in solids content caused the suspension to turn into a pulpy substrate, fulfilling the formal definition of a solid‐state fermentation [57]. The pH value was regulated by the automatic addition of 5 M NaOH with a peristaltic pump. Anaerobic conditions were maintained through constant nitrogen flow of 0.15 NL∙min^−1^, the exhaust gas was led over a cooling trap and the stirrer speed was adjusted to 200 rpm. The experiment was conducted in independent biological triplicates.

### 2.6 Quantitative cell determination

The biomass formation was estimated by the measurement of optical density at a wavelength of 600 nm (UV photospectrometer Lambda bio+, Perkin Elmer Corporation, Waltham, USA). Therefore, 0.1 g of cell suspension was mixed with 0.9 mL of MOPS buffer. This ‘quasi’ 1:10 dilution was further diluted to 1:100. As BSG particles settle down much faster than *C. uda* cells, the diluted fermentation broth was kept vibration‐free for 30 s before measuring the absorbance.

Additionally, the cell amount was calculated by cell count with a light microscope and a cell counting chamber (Neubauer improved 0.0025 mm^2^, Paul Marienfeld GmbH & Co. KG, Lauda‐Königshofen, Germany). A correlation for two biological duplicates, containing 5 w% BSG and 5 g∙L^−1^ yeast extract in aerobic 0.2 M MOPS buffer at pH = 7.4, was used for calculations in the other presented experiments.

### 2.7 Determination of enzymatic activity

*C. uda* has the capability of expressing cellulose and hemicellulose degrading enzymes [11, 12, 58]. The cellulase activity of the fermentation broth was measured by a filter paper assay as described previously for a general approach [59]. Nevertheless, the assay was adjusted to the requirements of the present process as follows: (I) 0.2 M MOPS buffer pH = 7.4 was applied instead of citrate buffer and (II) 6 cm^−2^ of filter paper (Whatman grade 1, Whatman plc, Little Chalfont, United Kingdom), 1 mL MOPS buffer and 1 mL of culture broth were incubated at 313 K and 1400 rpm for 240 min (Thermomixer‐compact, Eppendorf AG, Hamburg, Germany).

The xylanase activity was determined according to Rapp and Wagner [12] with some modifications: (I) 0.05 M sodium citrate buffer pH = 5 was applied instead of phosphate buffer in order to produce the 0.7 w% xylan testing solution and (II) 1 mL of culture broth and 1 mL of xylan solution were incubated at 323 K for 90 min.

The enzymatic reaction was stopped after the incubation period by heating up the sample to 368 K for 10 min. Sugar concentrations (cellobiose and xylose) were determined using HPLC, as described in the supporting information.

The enzymatic activity A, both for cellulase and for xylanase, was determined according to the Equation (2).

(2) $$\begin{array}{c} A=\frac{\left(c_{Sugar,Assay}-0.5\cdot c_{Sugar,Fermentation}\right)\cdot V_{Assay}}{M_{Sugar}\cdot t_{Assay}}\cdot 16.67 \end{array}$$

### 2.8 2D fluorescence spectroscopy

Samples of 0.2 mL of fermentation broth were pipetted into a 96 well plate (Black plate clear bottom with lid, Corning Inc., New York, USA) and put into a 2D fluorescence spectrometer (LS 55, Perkin Elmer Inc., Waltham, USA). The samples were stimulated at excitation wavelengths of 200–600 nm with a step size of 10 nm. The emitted light was detected in the range of 200–600 nm with an interval of 0.5 nm. The slit width was set to ‘seven’ both for excitation and emission, and the gain was adjusted to ‘medium’. Some of the resulting heat maps for both aerobic and anaerobic fermentations can be found in Figure S1. Calibration curves were generated for pure tryptophan and pyridoxin in a concentration range of 2–25 mg∙L^−1^, whereby the total peak volume was correlated with the concentration of the substance to be tested. Two emission maxima around Ex = 210–360 nm and Em = 350–480 nm were assigned to pyridoxine (aerobic fermentation), and one peak between Ex = 240–310 nm and Em = 300–450 nm was identified as tryptophan (anaerobic fermentation). The values, presented in this study, were calculated from the subtraction spectra between a fermentation sample and the spectrum of the initial fermentation medium before inoculation.

**FIGURE 1:**
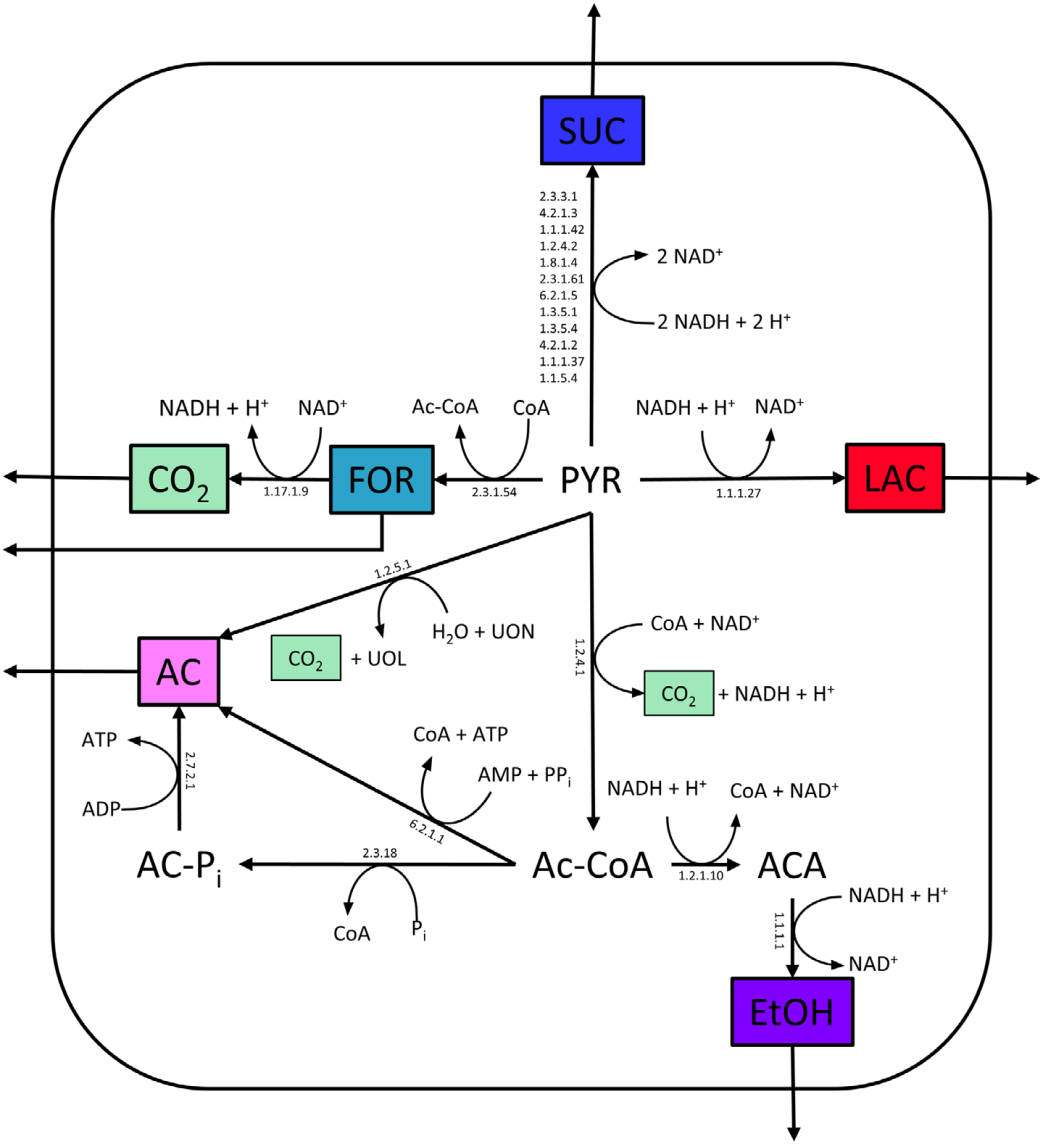
Potential central metabolism of *Cellulomonas uda*; AC, acetate; Ac‐CoA, acetyl coenzyme A; AT(D,M)P, adenosintri‐(di‐,mono‐)phosphate; CO_2_, carbon dioxide, CoA, coenzyme A; EtOH, ethanol; FOR, formiate; H_2_O, water; LAC, lactate; NAD^+^, nicotinamide adenine dinucleotide (oxidized); NADH, nicotinamide adenine dinucleotide (reduced); P_i_, phosphate; PP_i_, diphosphate; SUC, succinate; UOL, ubiquitol; UON, ubiquinone

The concentration of hydrolyzed protein in the fermentation supernatant was estimated by a conversion factor, which was calculated from the tryptophan content in barley protein as presented in literature (Equation 3) [60].

(3) $$\;c_{Barley\;protein}=\frac{1}{\chi_{Trp/Barley\;protein}}\;\cdot c_{Trp}$$

### 2.9 Biomass balance of the processes

In order to determine the extent of BSG degradation during the fermentation and the resulting cell accumulation, aerobic and anaerobic cultures were grown in biological duplicates as stated in Section 2.3. The pH was adjusted to 7.4 during operation but no samples were taken for analytical measurements because cells and BSG should remain in the fermentation flask.

At the end of the fermentation (14 days), the entire fermentation broth was centrifuged at 4200 × *g* for 45 min. This led to three phases: (I) a bottom BSG phase, (II) a middle phase containing the cells and (III) a top, liquid phase. The supernatant was discarded and the cell pellet was transferred to a separate vessel. Then, the bottom phase was washed with deionized water. This procedure was repeated three times. Afterwards, BSG and cells were determined gravimetrically after drying the fractions at 323 K for 48 h. In this way, a factor for the degradation of BSG was determined for aerobic, as well as for anaerobic fermentation conditions. In addition, the cell yield Y_X/S_ could also be calculated.

### 2.10 Feedstock determination

The composition of BSG was determined for the pressed feedstock as well as for BSG obtained from various time points of the fermentations. The contents of cellulose, hemicellulose, lignin as a sum of the acid‐soluble and ‐insoluble part, and ash were determined as described by the National Renewable Energy Laboratory (NREL) [61]: Cellulose and hemicellulose were determined by acid hydrolysis in pressure tight reaction vessels with a working volume of 100 mL (Pressure Plus, DWKLife Science GmbH, Mainz, Germany). Acid‐insoluble lignin and ash were determined gravimetrically after incineration to a constant weight in a muffle furnace (Model 301, Carbolite Gero Ltd, Sheffield, Great Britain). The portion of acid‐soluble lignin was measured by UV‐vis spectroscopy at 210 nm (Cary 60, Agilent Technologies, Santa Clara, USA), as described previously [62].

Furthermore, the protein content in BSG was determined by elementary analysis (vario Micro tube, Elementar Analysentechnik GmbH, Langenselbold, Germany) and calculated from the nitrogen content by a conversion factor of 5.7 [63]. Lipids were determined gravimetrically after extraction of 2–7.5 g of ground BSG for 4 h (∼30 cycles) with 200 mL of diethyl ether in a Soxhlet device (Normschliff Gerätebau GmbH, Wertheim, Germany) [64].

In order to calculate the absolute concentration of a specific BSG component, the percentage shares χ obtained from the NREL protocol were multiplied by the initial BSG concentration and the mass degradation factor that was obtained, as described in Section 2.9. The formula is shown in Equation (4). In case BSG samples were collected during operation (not only at the initial state and in the end of the fermentation), a linear depletion of biomass was assumed.

(4) $$\;c_{BSG\;component}=c_{BSG,0d}\cdot \chi_{BSG\;component}\;\cdot \frac{m_{BSG,14d}}{m_{BSG,0d}}$$

### 2.11 Determination of the higher heating value

The higher heating value (HHV) of BSG was calculated from the elementary composition as proposed in literature [65] according to Equation (5). The carbon and hydrogen contents were determined by elementary analysis and the oxygen content was assumed to be constant at χ_O_ = 34.37% [66].

(5) $$\begin{array}{ccc} HHV & = & -1,3675+0,3137\cdot \chi_{C}+0,7009\cdot \chi_{H}\\ & & +\;0,0318\cdot \chi_{O} \end{array}$$

### 2.12 BLAST

The Basic Local Alignment Search Tool (BLAST), which is available online at the website of the National Center for Biotechnology Information (NCBI, Rockville Pike, USA), was used to compare the genomes. Specific comparisons include the genes of the central metabolism of *C. uda*, with the genomes of *Cellulomonas gilvus* (CP002665.1), *Cellulomonas fimi* (CP002666.1) and *Cellulomonas flavigena* (NC_014151.1), as the Kyoto Encyclopedia of Genes and Genomes database (KEGG) included annotated genes for those close relatives of *C. uda*. Therefore, a nucleotide blast was performed between annotated genes of *C. gilvus*, *C. fimi*, *C. flavigena* and a shotgun sequence of *C. uda*, which is available at the NCBI (NZ_BJLP00000000.1). If significant similarities were detected (in this case: 80.21% < sequence identity < 93.29%), it was assumed that the related enzymes of the annotated genes in *C. gilvus*, *C. fimi* and/or *C. flavigena* could also be present in *C. uda*. The catalyzed reactions, including cofactor utilization, were adopted from the KEGG database and are categorized by the associated enzyme commission (E.C.) numbers, as shown in Figure 1. The reactions from pyruvate to succinate were visualized as one net reaction.

## 3 RESULTS AND DISCUSSION

### 3.1 Central metabolism of *C. uda*

The genomes of *C. uda* and *C. gilvus* contain about 3.5 M nucleotides and the sequence identity was calculated at 89.3% between them. *C. fimi* (*C. flavigena*) contains roughly 4.2 M (4.1 M) nucleotides and shows a sequence identity to *C. uda* of 84.7% (87.8%). Although *C. gilvus*, *C. fimi* and *C. flavigena* have already been identified as cognate organisms [67, 68], *C. uda* seems to be more closely related to *C. gilvus* than to the other organisms in terms of the complete genome.

As shown in Figure 1, identical findings were observed for the genes that code for the enzymes of the central metabolism. This figure shows potential conversion routes from pyruvate, the final metabolite of the Embden‐Meyerhof‐Parnas pathway, to the principal carbon‐containing metabolism end products succinate, lactate, formiate, acetate, ethanol and carbon dioxide, which have already been identified empirically for *C. uda* [58, 69]. The formate:NAD^+^ oxidoreductase (E.C.: 1.17.1.9) gene, which is responsible for formiate conversion to CO_2_, had the highest sequence identity to *C. fimi*. All the other presented genes showed the highest identity to *C. gilvus*.

It must be stated that the unidirectional reaction arrows do not necessarily represent a unidirectional character of the reaction. Most of the involved enzymes catalyze bidirectional reactions. For example, the ability to re‐assimilate products was reported for lactate in other *Cellulomonas* strains [15], and found for acetate in own experiments (data not shown). However, the reaction arrows represent the most probable direction under fermentative conditions.

### 3.2 Growth patterns of *C. uda* under aerobic and anaerobic conditions

**FIGURE 2:**
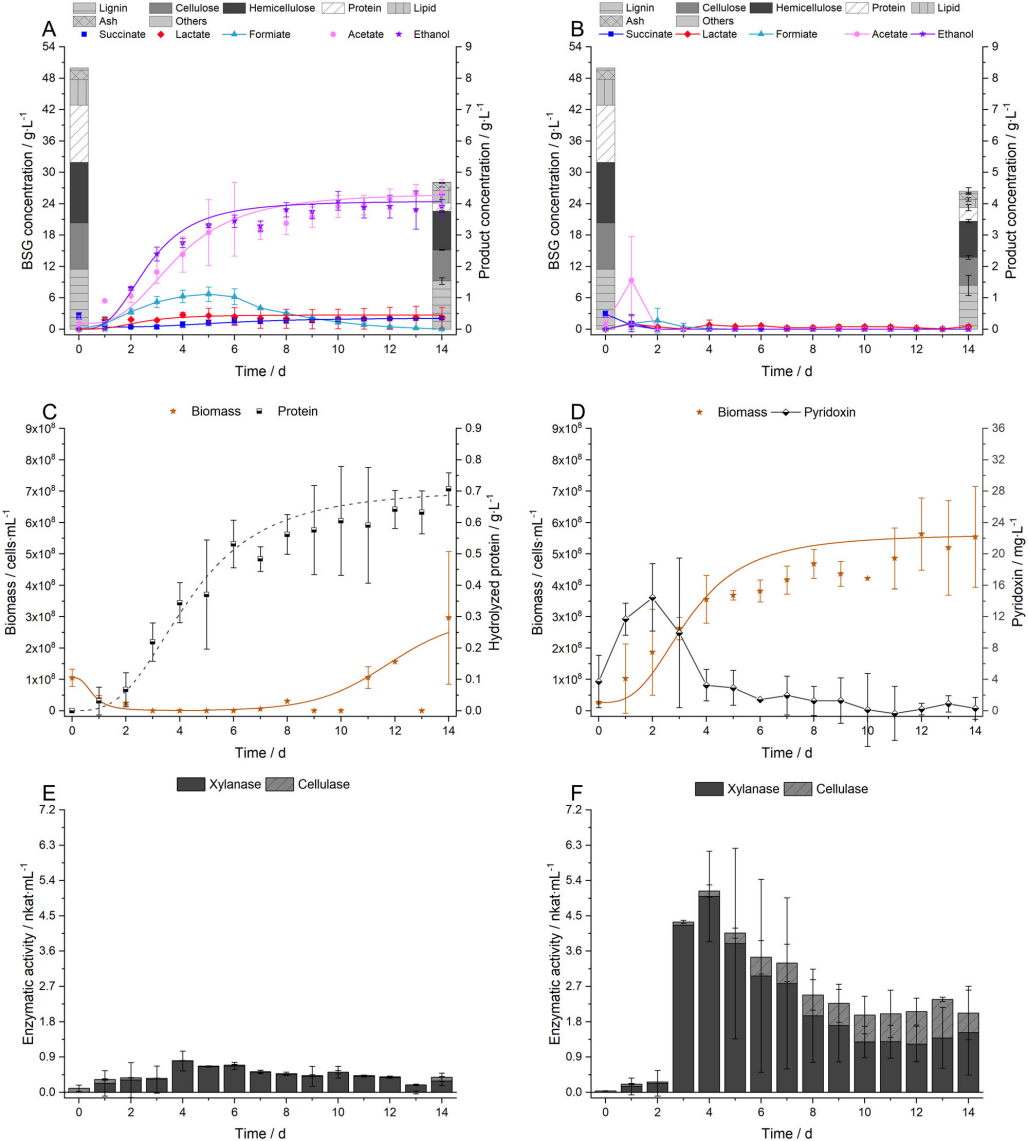
Fermentations with *C. uda* in a medium consisting of 5 w% ground BSG and 5 g∙L^−1^ yeast extract in 0.2 M MOPS buffer at pH = 7.4. A, C and E represent anaerobic conditions, B, D and F stand for aerobic conditions. (A,B) Product concentrations of succinate, lactate, formiate, acetate and ethanol during the fermentation. Substrate concentration classified by the components of BSG: lignin, cellulose, hemicellulose, protein, lipid, ash and other, minor components during the fermentation. (C,D) Biomass formation and hydrolyzed protein concentration or pyridoxin concentration during the fermentation. (E,F) Xylanase activity and cellulase activity. All data points were fitted by sigmoidal fits if the data followed this mathematical relation. The data show the mean values of two independent biological replicates; error bars represent the standard deviation

Figure 2 shows the growth of *C. uda* under aerobic and anaerobic conditions in terms of (subfigure in brackets): product formation and BSG consumption (A,B), cell growth (C,D) as well as cellulase and xylanase activity (E,F). Furthermore, the content of hydrolyzed BSG protein (C) is given for the oxygen‐free fermentation, and the concentration of pyridoxine (D) is demonstrated for the aerobic case.

In the anaerobic fermentation of BSG, *C. uda* produced five soluble products (succinate, lactate, formiate, acetate and ethanol) in a mixed‐acid fermentation, which have already been identified during its growth on cellulose [58]. Mainly acetate and ethanol were secreted into the fermentation broth in an equimolar ratio of 1.15 mol_EtOH_∙mol_Ac_^−1^. A similar ratio for those two metabolites was already described for the growth of *Cellulomonas *sp. (ATCC 21399) on cellulose [70]. The product yield was calculated as Y_P/S_ = 0.41 ± 0.03 g_Prod_^−1^∙g_BSG_^−1^. According to Figure 1, an equimolar production of CO_2_ occurs both for the formation of acetate and ethanol. If this CO_2_ formation is considered, the yield increases to Y_P+CO2/S_ = 0.73 ± 0.04 g_Prod+CO2_^−1^∙g_BSG_^−1^. The cell yield was calculated as Y_X/S_ = 0.11 ± 0.01 g_Cells_^−1^∙g_BSG_^−1^ according to chapter 2.9. *Cellulomonas* strains can oxidize formiate to CO_2_ in order to generate reduced NADH species [15]. If the decline in formiate concentration after 6 days of fermentation, as shown Figure 2A, is interpreted as the re‐assimilation and oxidation to CO_2_, the 16% gap in the carbon balance can be explained. However, no data on the maintenance metabolism of *Cellulomonadaceae* is available in literature. The BSG concentration decreased under oxygen‐free conditions from 50 to 28.1 ± 0.9 g∙L^−1^ as shown in Figure 2A, whereas the components were consumed at different proportions. The protein fraction was decreased by 85.7%, but 7.5% of degraded protein (6.5% of total protein) was not incorporated into the cell metabolism and instead accumulated in the fermentation broth. The lipid fraction was reduced by 51.2% and the cellulose and hemicellulose fractions were degraded by 32.8% and 23.2%, respectively. Lignin was only diminished to a minor extent (19.7%) as it does not contain nutritional value, but the break down might be necessary because the bacterial degradation of lignocellulosic substrates is always a synergistic process [71, 72, 73].

Under oxygen containing conditions, *C. uda* produced mainly biomass and did not secrete soluble products into the fermentation medium, which is in agreement with literature [58]. Sigmoidal increase of the cell density was seen for approximately 7 days and led to a total cell yield of Y_X/S_ = 0.22 ± 0.05 g_Cells_^−1^∙g_BSG_^−1^, which was basically twice the anaerobic cell yield. When referring to the yield of the consumed carbohydrates, it increased to Y_X/CH_ = 0.64 ± 0.14 g_Cells_^−1^∙g_Carbohydrates_^−1^, which was slightly higher than previously reported for *Cellulomonas biazotea* with Y_X/CH_ ≈ 0.5 g_Cells_^−1^∙g_Carbohydrates_^−1^ [9]. To verify the given cell concentrations, fermentation samples of aerobic and anaerobic conditions were investigated with light‐ and confocal laser scanning microscopy, respectively. The captured images can be seen in Figure S2 and demonstrate a higher cell density in the oxygen‐containing environment. Furthermore, a correlation between cell concentration C, determined by cell count, and optical density was established according to Equation (6).

(6) $$C=OD_{600nm}\;\cdot 10^{7}$$

Since almost no liquid products were produced, the product yield was determined with Y_P/S_ = 0.004 ± 0.003 g_Prod_^−1^∙g_BSG_^−1^ in the aerobic case. It was reported that *Cellulomonas gelida* UQM 2480 produces 81.6 times more CO_2_, if the gas phase holds an oxygen concentration of 21% compared to (quasi) anaerobic conditions with an oxygen concentration < 0.1% [13]. Considering this increased CO_2_ production in contrast to the anaerobic fermentation presented in this study, the product yield increases to Y_P+CO2/S_ = 1.11 ± 0.16 g_Prod+CO2_^−1^∙g_BSG_^−1^. BSG concentration decreased from 50 to 26.1 ± 1.9 g∙L^−1^ in the aerobic case, whereas the components were affected at different proportions by bacterial degradation. The protein fraction was again the highest consumed fraction with a proportion of degradation by 76%. To our knowledge, the massive protein depletion in lignocellulosic biomass initiated by *C. uda*, both under presence and absence of oxygen, has not been reported in literature so far. Lipids, cellulose, hemicellulose and lignin were degraded by 68.5%, 39%, 40.1% and 27.1%, respectively. In general, carbohydrates were slightly higher consumed under aerobic than under anaerobic conditions.

### 3.3 Enzymatic activity during aerobic and anaerobic growth of *C. uda*

In most papers dealing with *Cellulomonas* strains, either the cellulase/glucanase activity [9, 11, 74] or the xylanase activity [12, 14, 75, 76] is presented. However, holistic approaches in terms of various enzyme classes are reported too [18, 77]. In this study, the anaerobic enzymatic activity was quite low at A_Cell,max_ = 0.10 ± 0.02 nkat∙mL^−1^ and A_Xyl,max_ = 0.80 ± 0.25 nkat∙mL^−1^, respectively. The activities increased for aerobic conditions to A_Cell,max_ = 0.98 ± 0.05 nkat∙mL^−1^ and A_Xyl,max_ = 5.00 ± 1.14 nkat∙mL^−1^, respectively. The enzyme activity of *Cellulomonas* sp. B6 was reported to be approximately 3.3 nkat∙mL^−1^ for cellulase and 10.0 nkat∙mL^−1^ for xylanase when grown aerobically on lignocellulosic substrates like sugar cane straw or extrusion‐pretreated wheat straw [77], which is in accordance with the observations made in the present study.

On one hand, the enzymatic activity in general was found to be lower when anaerobic conditions were applied instead of an oxygen‐containing gas phase, which was already reported for *C. uda* grown on cellulose [58]. On the other hand, the xylanase activity was higher than the cellulase activity both in aerobic and anaerobic conditions. This effect has already been described for *C. flavigena* NIAB 441 and various lignocellulosic substrates, like wheat straw, grass straw or bagasse. It was reported in literature that xylanase activity can only be diminished by a factor of 3–6 (but not completely shut down) if xylan‐free substrates like carboxymethylcellulose, cotton wool, cotton stalks or filter paper are applied [5].

It was surprising that xylanase activities decreased again at later time points of the experiment, which can best be observed in Figure 2F. It was already reported, that the presence of xylan can inhibit cellulose degradation, both for technical enzyme mixtures [78] and for genetically‐engineered *Saccharomyces cerevisiae* cells, which have the ability to metabolize cellobiose [79]. Furthermore, many enzymes can be inactivated by physical interaction with lignocellulosic materials [80]. Activity losses of the enzymes, as described above, might be triggered by comparable effects and have already been observed for cellulase activity in other *Cellulomonas* species [8].

### 3.4 Fluorophores during aerobic and anaerobic growth of *C. uda*

Four key metabolic fluorophores are described in literature: tryptophan, riboflavin, NAD(P)H, pyridoxine along with derivates like pyridoxamine and pyridoxal‐5′‐phosphate. More important biogenic fluorophores are the amino acids tyrosine and phenylalanine [81, 82]. In fermentations with lignocellulosic substrates, humic and fulvic acids derived from lignin can be fluorophores too [83]. For the fermentations done in this study, pyridoxine was exclusively detected at the beginning of the aerobic fermentation, and tryptophan (indicating hydrolyzed BSG protein) was only seen in the anaerobic fermentation. Both signals showed identical or even increased intensity in the filtered supernatant compared to the ‘raw’ fermentation broth, containing cells and BSG particles.

Pyridoxin plays an important role in the amino acid metabolism of many organisms, as already shown for Escherichia* coli* K12 [84]. The importance can also be demonstrated using the following examples: (I) In *S. cerevisiae* RTY110/pRB58, the intracellular pyridoxine concentration can be correlated to metabolic activity and exponential cell growth [81]. (II) The respiration rate of aerobically grown Saccharomyces* carlsbergensis 4228* can be increased by a factor of 3.5, if pyridoxine is added to the fermentation broth [85]. (III) Pyridoxine is an important co‐factor in the Krebs cycle [86]. (IV) Pyridoxal‐5′‐phosphate, which can be synthesized from pyridoxine, is a cofactor for the synthesis of 5‐aminolevulinic acid. In turn, 5‐aminolevulinic acid is the precursor for the synthesis of various tetrapyrroles in bacteria [87]. These molecules play an important role as cofactors for hem‐containing enzymes located in the respiratory chain. In this context, one of the most important proteins is cytochrome C, which was already detected in the membrane of *C. fimi* [19]. Since the fluorescence signal of pyridoxin was found in the culture supernatant, it seems likely that *C. uda* extracts pyridoxine from the BSG and takes it up in a second step in order to gather cofactors for amino acid synthesis and/or cell respiration. However, since pyridoxin has exclusively been detected in aerobic processes, there is some evidence that pyridoxin is related to cell respiration. Nevertheless, the pyridoxine concentration in BSG was described as 0.7 ppm [88], which is much lower than determined in this study.

Many soil bacteria are capable of secreting extracellular proteases, because the N‐availability in their habitat is mostly limited to protein‐derived sources [89, 90]. This includes *Cellulomonas* strains, which are able to produce (temperature‐ and pH‐resistant) proteinases, e.g. Cellulomonas* bogoriensis* [91] or *Cellulomonas* sp. ATCC 21399 [16]. On one hand, utilization is possible through direct uptake of amino acids/peptides, whereas the peptides serve as a nitrogen‐, carbon‐ and energy source. On the other hand, the uptake is feasible after deamination (e.g. hydrolytic, reductive or oxidative), and uptake in the form of ammonia or nitrate (after nitrification of NH_4_^+^). In the second case, only the nitrogen demand is served. This strategy applies if the carbon supply is high (e.g. aerobic conditions) because the expression of an amino acid oxidase and a NH_4_^+^ transporter is more economic than the expression of energy‐dependent transporters for various amino acids and di‐ and oligopeptides. Moreover, the carbon and energy supply by amino acids is not mandatory due to high carbohydrate acquisition [3, 92–94]. The amino acid deamination by an amino acid oxidase has already been described for Cellulomonas* cellulans* AM8 [95]. Furthermore, the release of NH_4_^+^ into the medium was measured for *C. uda* grown in a peptone containing medium, which is a strong indication of the production of an extracellular amino acid oxidase [6]. During the aerobic fermentation, the entirety of amino acids and peptides was probably consumed by one of the aforementioned mechanisms; this is since no tryptophan fluorescence was observed (Figure S1E) but the protein content in BSG was degraded to a high percentage (chapter 3.2). Vice versa, in the anaerobic fermentation, tryptophan or peptides containing tryptophan, accumulated in the fermentation broth, which correspond to a protein concentration of approximately 0.7 g∙L^−1^ (verified by Bradford assay, data not shown). This might point to the lack or diminished expression of an amino acid oxidase. This could be induced by low carbon availability because the cellulase‐/xylanase activities were lower, which resulted in a lower degradation of structural carbohydrates.

In both fermentation scenarios an additional emission maximum was built up in the range of Ex = 320–420 nm, Em = 400–540 nm, which was described as fulvic acid‐like fluorescence (Figure S1E, F) [96]. Many soil bacteria are able to degrade lignin using peroxidases or laccases [97]. In particular, *Cellulomonas* sp. can secrete a manganese peroxidase [98], which is one of the most common lignin‐degrading enzymes [99]. It seems likely that this broad emission maximum, which is also higher in the aerobic case, derives from the degradation of lignin. Also, lignin was diminished to a higher share in the solid BSG residue of the aerobic fermentation (chapter 3.2). However, no proof could be made as calcium‐lignosulphonate and fulvic acid showed different fluorescence patterns than the fermentation samples (Figure S3).

### 3.5 Optimization of the anaerobic process

**FIGURE 3:**
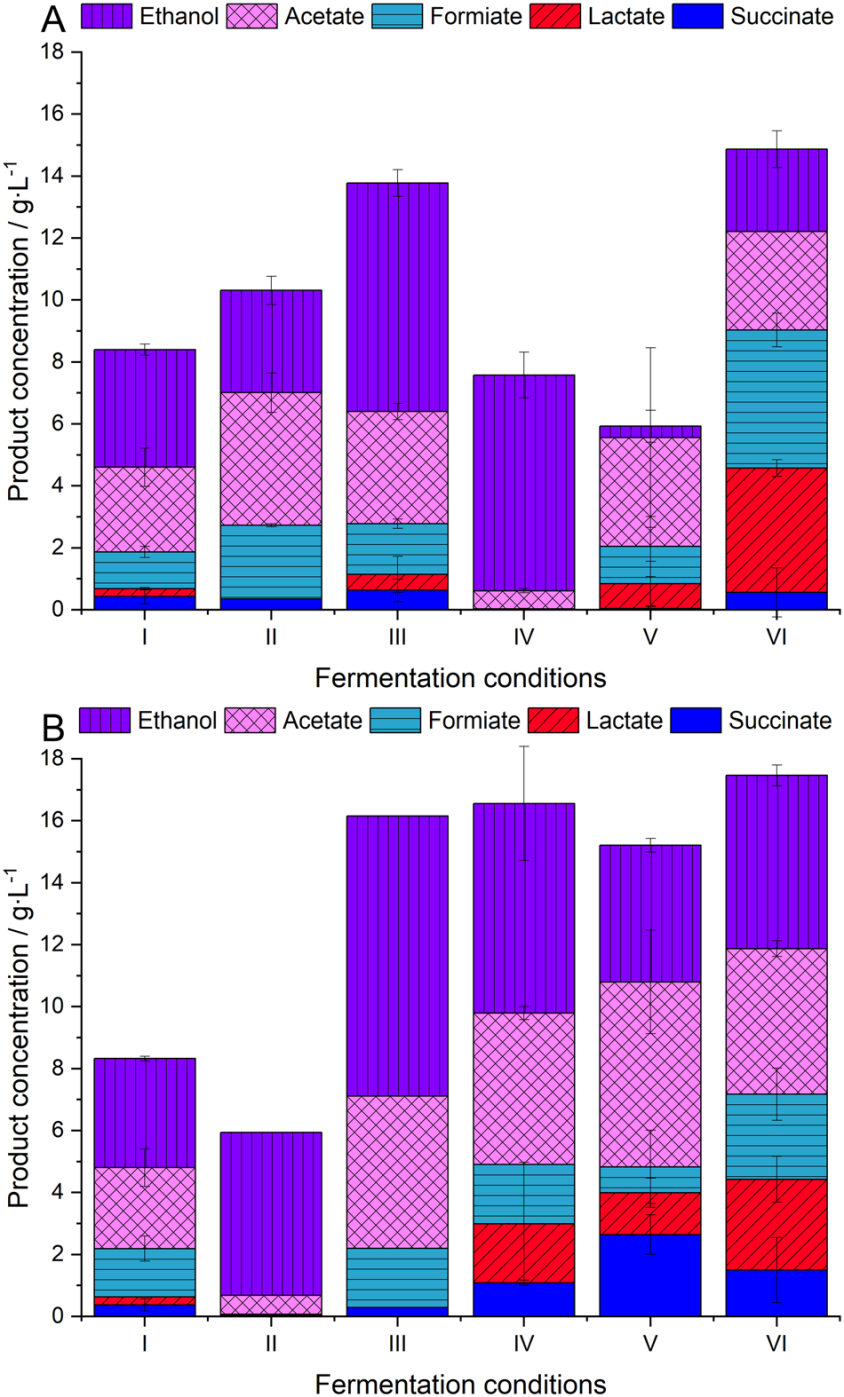
Final product concentration of various fermentations with *C. uda* in terms of succinate, lactate, formiate, acetate and ethanol. (A) (I) Reference fermentation with anaerobic 0.2 M MOPS buffer, 5 w% of ground BSG, YE at pH = 7.4; (II) addition of K_3_FeCN_6_ and YE at pH = 7.4; (III) addition of cys‐HCl and YE at pH = 7.4, kinetic shown in Figure 4A; (IV) addition of cys‐HCl and YE at pH = 5, kinetic shown in Figure 4B; (V) addition of cys‐HCl and YE at pH = 9; (VI) BSG that was not pressed + addition of cys‐HCl and YE at pH = 7.4, kinetic shown in Figure 4C. (B) (I) Reference fermentation with anaerobic 0.2 M MOPS buffer, 5 w% of ground BSG, YE at pH = 7.4; (II) OS‐treated BSG at pH = 7.4; (III) OS‐treated BSG + addition of cys‐HCl and YE at pH = 7.4. In the remaining fermentations, cys‐HCl and YE were always applied in the reported concentration, the pH was adjusted to 7.4. Additionally, the following enzymes were added at the fifth day of the fermentation: (IV) cellulase, kinetic shown in Figure 4D; (V) hemicellulase; (VI) cellulase + hemicellulase. The shown values represent the mean value of two independent biological replicates after 14 days of fermentation (except subfigure B, fermentation II and III); error bars represent the standard deviation. OS, organosolv; YE, yeast extract

**FIGURE 4:**
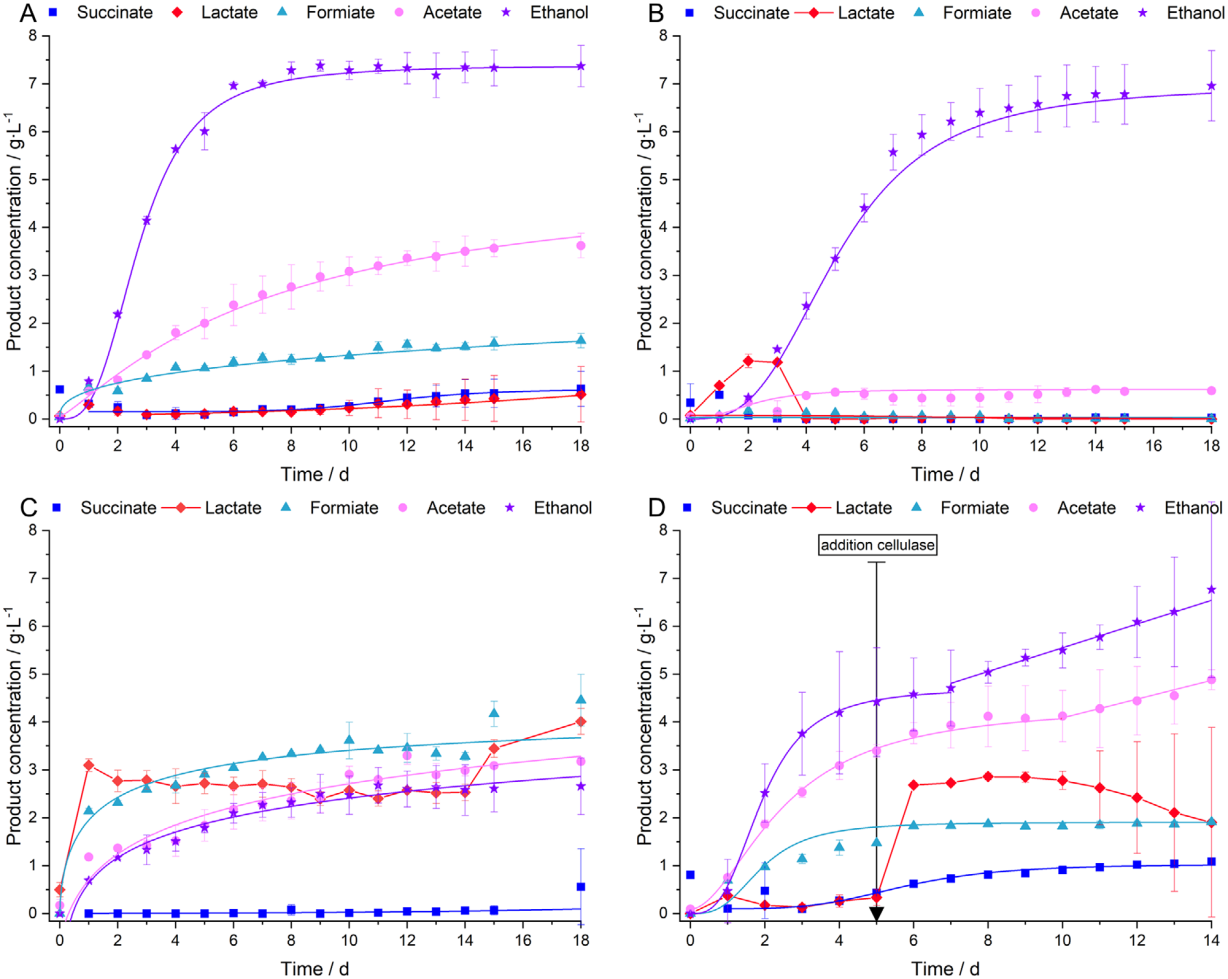
Product formation kinetics of fermentations with *C. uda* in anaerobic 0.2 M MOPS buffer and 5 w% of ground BSG in terms of succinate, lactate, formiate, acetate and ethanol. (A) Addition of cys‐HCl and YE at pH = 7.4; (B) addition of cys‐HCl and YE at pH = 5; (C) BSG that was not pressed + addition of cys‐HCl and YE at pH = 7.4; (D) addition of cys‐HCl and YE at pH = 7.4 + cellulase supplementation on day 5. The data show the mean values of two independent biological replicates; error bars represent the standard deviation. Data points of succinate‐, formiate‐, acetate‐ and ethanol‐formation were fitted by sigmoidal fits (or by a mixture of sigmoidal and linear fits in subfigure D), lactate data points were not fitted since no suitable mathematical description was possible; YE, yeast extract

Figure 3 shows the final product concentrations for various fermentation options of the anaerobic fermentation, as presented in section 3.2. Figure 3A,B represents two separate fermentation runs, from which no significant deviations were found for the reference experiments (each shown at position (I)). To simplify, the fundamental fermentation kinetics are only presented for some representative experiments in Figure 4. In general, deviations between biological replicates were smaller in terms of product concentrations than for other data presented before (e.g. in Figure 2C–F). This was probably caused by the liquid character of the samples used for product analytics in contrast to the suspension samples (with higher inhomogeneity) applied for fluorescence measurements, biomass determination and enzyme assays.

It was already found from literature that the oxidoreduction potential can direct the product spectrum in mixed acid fermenting of *E. coli* K12 [100], promote the production of 1,3‐propanediol in *Klebsiella pneumoniae* [101], enhance the formation of aroma compounds from amino acids in *Lactococcus lactis* [102], increase the formation of citric acid in *Aspergillus niger* [103] and activate the expression of enzymes in *Yarrowia lipolytica* [104]. Because of this huge physiological impact, oxidizing (K_3_FeCN_6_) and reducing agents (cys‐HCl) were added to the fermentation broth. Oxidizing agents increase the intracellular NAD(P)^+^/NAD(P)H ratio [101, 105]. Therefore, oxidized compounds like acetate and formiate were produced in slightly higher concentrations (Figure 3A (II)) compared to the reference fermentation, shown in Figure 3A (I). On the contrary, a low oxidoreduction potential, as applied in the fermentation shown in Figure 3A (III), decreases the intracellular NAD(P)^+^/NAD(P)H ratio. Therefore, reduced—NAD(P)^+^ regenerating—products were favoured, which applied mainly to ethanol in this experiment. Compared to the reference fermentation, the ratio of ethanol to acetate was increased from 1.08 to 1.59 mol_EtOH_∙mol_Ac_^−1^. Similar observations concerning the ethanol/acetate ratio were also reported for different *Clostridium* strains [106].

The favourable reducing conditions were tested with lowered pH = 5 and increased pH = 9, as shown in Figure 3A (IV) and (V), respectively. For pH = 5, the final ethanol concentration and productivity were comparable to pH = 7.4 (Figure 4A,B), but the ethanol/acetate ratio was significantly improved to 9.2 mol_EtOH_∙mol_Ac_^−1^. However, the question arises on which ATP generating mechanisms occur during anaerobic fermentation without acetate formation. Vice versa, the increase to pH = 9 lowered the ethanol/acetate ratio to 0.08 mol_EtOH_∙mol_Ac_^−1^. The pH‐dependency (and induced cellular stress) on the ratio of solventogenesis to acidogenesis, which was observed in these experiments for *C. uda*, is mainly known for *Clostridiaceae* [106, 107, 108]. In conclusion, a process starting at pH = 7.4 (growing cells) and a subsequent shift to pH = 5 (growth‐uncoupled ethanol production), seemed to yield interesting results. However, the sudden pH drop led to cell inactivation, which became apparent because the formation of products stopped immediately and sugar (cellobiose, xylose, arabinose) accumulated in the fermentation broth (Figure S4).

Furthermore, BSG was subjected to OS‐treatments before the fermentation as these treatments extract mainly lignin from wooden biomass [54, 109]. The removal of lignin should result in facilitating the bacterial degradation of structural carbohydrates. However, the growth with pure OS‐pretreated BSG showed a diminished product formation (Figure 3B (II)). If yeast extract and cys‐HCl were applied (Figure 3B (III)), no significant increase in the product formation was observed in comparison to similar conditions with untreated BSG (Figure 3A (III)).

The addition of cellulases, hemicellulases or a combination of both enzyme classes, as shown in Figure 3B (IV) to (VI), did not significantly alter the total product concentration either. It was observed that the lactate concentration in these cultures increased immediately after the addition of the enzymes, as exemplarily shown in Figure 4D. These results show that the addition of nutrients and the adjustment of the redox environment seem to have a greater influence on product concentrations than facilitated carbohydrate degradation by OS‐treatments or the addition of technical enzymes.

Similar results in terms of lactate formation were also observed, when soluble sugar was present at the beginning of the fermentation (Figure 4C and Figure 3A (VI)). This is because adherent molecules like glucose, maltose and maltotriose were not removed from the BSG by pressing [55, 69]. Increased lactate formation, can be an indication of an excess in carbohydrates and resulting energy dissipation by different mechanisms like futile cycles, energy uncoupling, metabolic shifts or overflow metabolism [110, 111, 112]. For example, increased lactate production was already observed for *Klebsiella aerogenes* (excess in glucose) [113] and *Clostridium cellulolyticum* (excess in cellobiose) [114]. In this study, the sudden appearance of additional low‐chain length sugar (e.g. induced by enzyme addition) might exceed the catalytic capability of the enzymes involved for the production of acetate and ethanol. Therefore, a metabolic backlog might lead to the accumulation of glycolysis intermediates. These intermediates might act as inducers for increased lactate production, as reported for fructose‐1,6‐diphosphate, which is known to activate lactate dehydrogenase tetramers with high activity in *Streptococcus bovis* [115].

Finally, the question arises as to which of the optimization approaches are most promising. The economical application of the pretreatment strategies is complicated since OS‐treatments require an excessive energy demand [116] and technical enzyme mixtures have high manufacturing costs [20]. In conclusion, the minor increases in product concentration and concomitant diversification of the product spectrum, e.g. by lactate formation, do not justify the application of these treatments. Therefore, reducing conditions in 0.2 M MOPS buffer with 5 g∙L^−1^ yeast extract at pH = 7.4 and sugar‐free BSG seem to be the most suitable in achieving significant product concentrations. If only the production of ethanol is desired, a reduction of the pH value to 5 is recommended.

### 3.6 Solid‐state fermentations in a bioreactor

The most advantageous conditions identified in the suspension cultures in flask‐scale (Section 3.5) were also investigated on a reactor scale, which made it possible to take solid samples during operation even though the solids content was increased to 15 w%.

**FIGURE 5:**
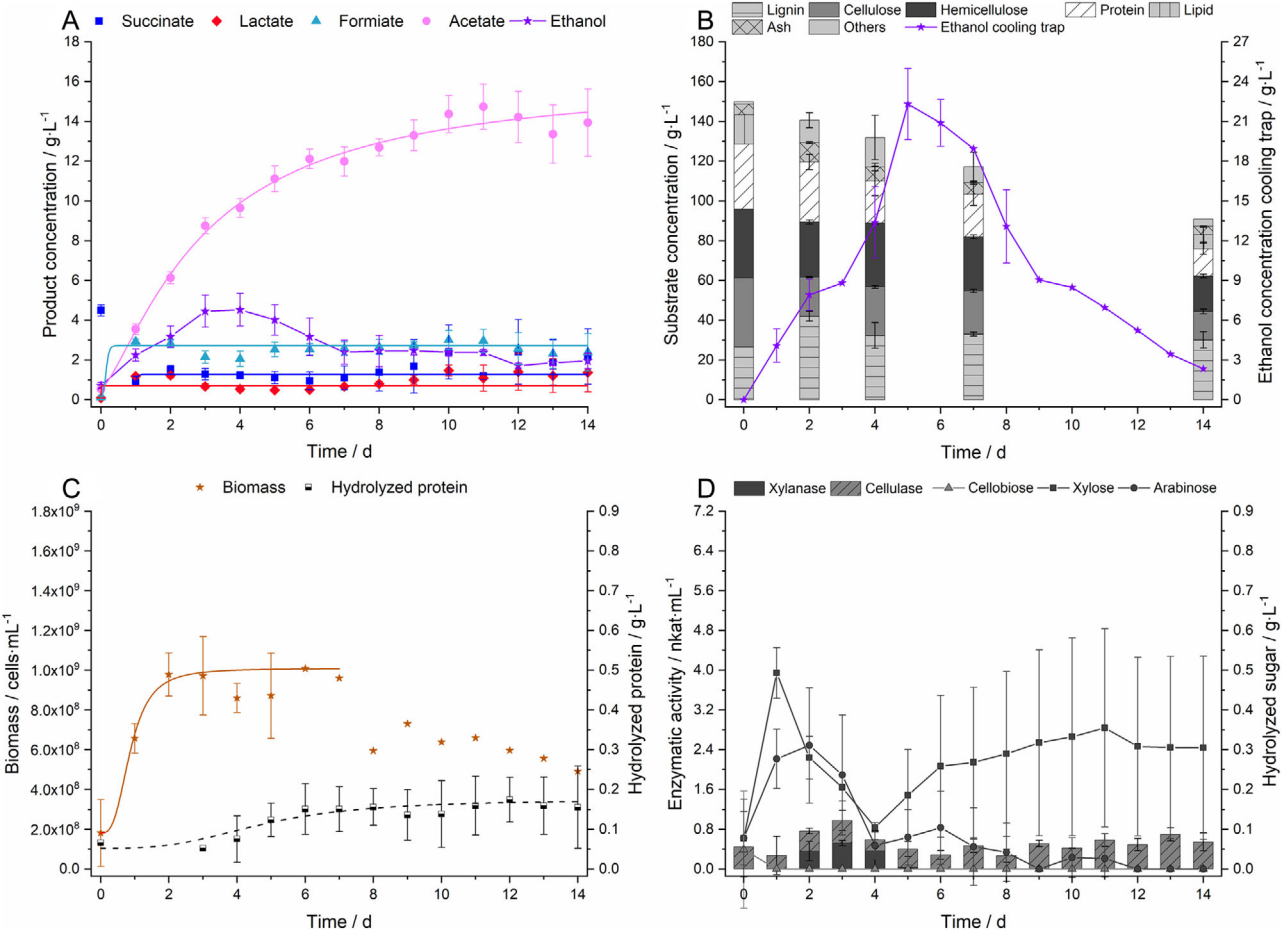
Fermentations with *C. uda* in anaerobic 0.2 M MOPS buffer and 15 w% of ground BSG, 15 g∙L^−1^ yeast extract and 1 g∙L^−1^ cys‐HCl. (A) Product formation in terms of succinate, lactate, formiate, acetate and ethanol; (B) BSG consumption, consisting of lignin, cellulose, hemicellulose, protein, lipid, ash and other, minor components as well as ethanol concentration in the cooling trap; (C) biomass formation and hydrolyzed protein concentration; (D) xylanase activity and cellulase activity and released sugar due to bacterial degradation: cellobiose, xylose and arabinose. The data show the mean values of three independent biological replicates with one exception: the cell concentration, starting at day 6, shows single determinations as the cells stuck to the stirrer in two of three replicates; error bars represent the standard deviation. All data points were fitted by sigmoidal fits if the data followed this mathematical relation

The main product in this fermentation was acetate with a final concentration of 14.7 ± 1.14 g∙L^−1^ (Figure 5A), which was four times the concentration compared to the fermentation with 5 w% of BSG, as shown in Figure 4A. Assuming the ethanol/acetate ratio of 1.59 mol_EtOH_∙mol_Ac_^−1^ as determined in chapter 3.4, an ethanol concentration of approximately 18.3 g∙L^−1^ was expected. However, only a maximum concentration of 4.53 ± 0.82 g∙L^−1^ was observed in the fermentation broth. The low boiling point of ethanol of 351 K led to an accumulation of ethanol in the cooling trap to a maximum concentration of 22.3 ± 2.68 g∙L^−1^ (Figure 5B). It can be observed that both the ethanol titre in the fermentation broth and in the cooling trap decreased from some point on likely due to constant nitrogen flow, which was mandatory to obtain anaerobic conditions in the reactor. On one hand, ethanol evaporation is very undesirable in lab‐scale. On the other hand, this unfavourable effect could be used to enrich ethanol by gas stripping in a potential production process [106]. Stripping is a preferable purification method for ethanol from a fermentation broth as distillation is quite costly (in particular for low ethanol contents) and only economically feasible for ethanol concentrations > 5 w% [106, 117, 118].

Apparently, cellulose and hemicellulose were consumed concomitantly (Figure 5B). This is in accordance with literature, where no diauxic growth was reported for the growth of *C. uda* in a medium containing glucose and xylose [6]. The protein content degraded a substantial amount (57.9%) and the lignin content decreased a minor amount (degradation by 12.5%). The proportions of cellulose, hemicellulose and lipids were decreased by 46.1%, 48.9% and 52.3%, respectively.

The cell concentration followed a sigmoidal trend as shown in Figure 5C, probably due to the aerobic character of the preculture. The aerobic preculture also affected further aspects, as follows: (I) The concentration of accumulated protein was only 0.17 ± 0.06 g∙L^−1^ and therefore decreased by 75.3% compared to a fermentation, without an aerobic preculture (Figure 5C vs. Figure 2C). (II) A basal cellulase activity was maintained during the entire fermentation whereas the xylanase activity could only be determined between day 2 and 5 of the fermentation (Figure 5D), which seems to be in contrast to common references [58]. Low concentrations of xylose and arabinose were measured through the entire process—but no cellobiose accumulated. This might point to an adaption on cellobiose as a preferred substrate in the preculture, which was not altered in the anaerobic main culture.

### 3.7 Holistic usage of all occurring streams

In terms of a biorefining concept, holistic approaches for all occurring streams are required: (I) The liquid portion of the BSG stream removed by pressing, can potentially be used as a basis for a fermentation medium with the purpose of lactate production (final titres of approximately 80 g∙L^−1^) [55], where the product titres can be estimated from the ingredients of the liquor in advance by means of kinetic modelling [119]. (II) Ethanol can be separated from the fermentation broth, e.g. by gas stripping, as stated in chapter 3.6. (III) Liquid by‐products like acetate, can be further converted to methane with organisms used for wastewater purification (e.g. contained in activated sludge) [106]. Therefore, the separation of acetate from the fermentation broth could be performed by an additional pressing step. By‐products of the fermentation should not be a hindrance to the methanogenesis as the production of methane was already described from acetate, formiate and a mixture of CO_2_/H_2_ in an anaerobic digestion (AD) approach [120] (IV) Subsequently, *C. uda* biomass could be separated from BSG by a centrifugation step (chapter 2.9) and applied as a source of single cell protein. (V) Finally, the only remaining stream—the solid BSG residue of the fermentation—can be used to generate energy, since the HHV of BSG from the fermentation presented in Figure 5 did not decrease during the fermentation, as can be seen from the following values: 19.66 MJ∙kg^−1^ (untreated BSG), 18.93 ± 0.3 MJ∙kg^−1^ (2 day fermentation), 18.63 ± 0.56 MJ∙kg^−1^ (4 day fermentation), 19.17 ± 0.42 MJ∙kg^−1^ (7 day fermentation) and 19.72 ± 0.49 MJ∙kg^−1^ (14 day fermentation). Comparable HHVs were reported for other lignocellulosic biomasses [121]. Therefore, the described fermentation can be embedded into a holistic application of all occurring BSG streams.

## 4 CONCLUDING REMARKS

In this study, the application of BSG, an abundant by‐product of the brewing process, was demonstrated as feedstock for fermentations in suspension cultures and solid‐state fermentations using the organism *C. uda*.

Aerobic fermentations led to the formation of cell biomass and increased the expression of cellulose‐ and hemicellulose degrading enzymes. In anaerobic fermentations, valuable liquid products like acetate and ethanol were produced. Therefore, the production of single cell protein/enzymes or bulk chemicals from BSG gives two potential applications within one organism. Moreover, these fermentations were used in order to develop various strategies for process analytics: (I) In terms of process engineering, a strategy was obtained to maintain a constant pH in flask‐scale experiments. (II) Rapid, non‐invasive 2D fluorescence measurements gave insights into the growth phase of the organism by measuring pyridoxine (aerobic) and tryptophan (anaerobic). (III) A correlation between cell number and optical density was determined, which has not been described for fermentations with solid BSG so far and provides an easy opportunity to determine the cell concentration.

The variation of media compositions allowed comparisons to be made to the central metabolism of the organism, which was visualized for the first time based on gene homologies to close relatives of *C. uda*. Furthermore, the optimization of the anaerobic process showed effective results, which were already reported for other bacteria genera but not for *C. uda*: (I) Reducing conditions promote the formation of ethanol. (II) Lower pH values decrease side product formation while maintaining ethanol production. (III) High availability of low chain length sugars promote the formation of lactate probably due to an overflow metabolism.

However, if the proposed process is put into the context of a holistic biorefinery, increasing product titres will be mandatory in order to gain an economically feasible process: In terms of final titres this approach cannot compete with more established microorganisms (especially in terms of ethanol production). However, advantages arise as a single organism can be applied in a consolidated bioprocess, which can degrade lignocellulosic biomass and convert it to valuable products in one reaction step. Furthermore, it may be assumed that product titres could be further increased by genetically engineered *C. uda*, where one‐enzyme conversions (e.g. from pyruvate to lactate or formiate) could possibly be knocked out quite easily or by further elevating the substrate loading in the fermentation. Therefore, this study proposes an interesting opportunity for the biotechnological application of the solid parts of BSG at mild process conditions. In addition, the introduction of various process monitoring and regulation strategies will support future work in the optimization of solid‐state fermentations with different feedstock and/or organisms; a rapidly expanding area of research in today's world.

## NOMENCLATURE

**Table:** 

A	[nkat∙mL^−1^]	Cellulase‐/xylanase activity
A_Cell,max_	[nkat∙mL^−1^]	Maximum cellulase activity
AD	[‐]	Anaerobic digestion
A_Xyl,max_	[nkat∙mL^−1^]	Maximum xylanase activity
C	[Cells∙mL^−1^]	Cell concentration
c_Barley protein_	[g∙L^−1^]	Concentration of barley protein
c_BSG component_	[g∙L^−1^]	Concentration of a BSG component
c_BSG,0d_	[g∙L^−1^]	BSG concentration at the beginning of the fermentation
c_Sugar,Assay_	[g∙L^−1^]	Sugar concentration after enzyme assay
c_Sugar,Fermentation_	[g∙L^−1^]	Sugar concentration in fermentation broth
c_Trp_	[g∙L^−1^]	Tryptophan concentration
Em	[nm]	Emission wavelength
Ex	[nm]	Extinction wavelength
m_BSG,0d_	[g]	Mass of BSG at the beginning of the fermentation
m_BSG,14d_	[g]	Mass of BSG at the end of the fermentation
mol_Ac_	[mol]	Moles of acetate
mol_EtOH_	[mol]	Moles of ethanol
M_Sugar_	[g∙μmol^−1^]	Molecular weight of sugar
NL	[L]	Normliter
nm	[‐]	Nanometre
p	[bar]	Pressure
pH_current_	[_‐_]	Current, measured pH
pH_target_	[_‐_]	Target pH of the fermentation
px	[‐]	Pixels
t_Assay_	[min]	Time span of enzyme assay
V_Assay_	[mL]	Volume of enzyme assay
V_Culture,current_	[mL]	Current volume of the fermentation broth
V_NaOH_	[mL]	Volume of 5 M NaOH addition
vol%	[v/v]	Volume percent
w%	[w/w]	Weight percent
Y_P/S_	[g_Prod_^−1^∙g_BSG_^−1^]	Liquid product/substrate (BSG) yield
Y_P+CO2/S_	[g_Prod+CO2_^−1^∙g_BSG_^−1^]	Liquid + gaseous product/substrate (BSG) yield
yr	[‐]	year
Y_X/CH_	[g_Cells_^−1^∙g_Carbohydrates_^−1^]	Cell/carbohydrate yield
Y_X/S_	[g_Cells_^−1^∙g_BSG_^−1^]	Cell/substrate (BSG) yield
χ_BSG component_	[w%]	Share of a BSG component
χ_C_	[w%]	Share of carbon in BSG
χ_H_	[w%]	Share of hydrogen in BSG
χ_O_	[w%]	Share of oxygen in BSG
χ_Trp/Barley protein_	[w%]	Share of tryptophan in barley protein

## CONFLICT OF INTEREST

The authors have declared no conflicts of interest.

## Supporting information



Supporting Information

## Data Availability

Data available on request from the authors.
