# Supplementary material for: Optimization of bioprocesses with Brewers’ spent grain and Cellulomonas uda
Source: Eng Life Sci. 2021 Aug 27;22(3-4):132–51. doi: 10.1002/elsc.202100053 (PMC8961044; doi:10.1002/elsc.202100053)
Supplement: Supplementary file 1 — Supporting Information [file ELSC-22-132-s001.docx]

Supporting Information to Research Article

**Optimization of bioprocesses with Brewers’ spent grain and *Cellulomonas uda***

Alexander Akermann^1^

Jens Weiermüller^1^

Jonas Nicolai Chodorski^1^

Malte Jakob Nestriepke^1^

Maria Teresa Baclig^1^

Roland Ulber^1*^

^1^TU Kaiserslautern, Department of Mechanical and Process Engineering, Chair of Bioprocess Engineering, Gottlieb-Daimler-Straße 49, 67663 Kaiserslautern, Germany

**Correspondence:** Prof. Dr. Roland Ulber (e-mail: [ulber@mv.uni-kl.de](mailto:ulber@mv.uni-kl.de), tel: +49631205‑4043/‑4044, fax: +49 631 205-4312).

Department of Mechanical and Process Engineering, Chair of Bioprocess Engineering, Gottlieb-Daimler-Straße 49, 67663 Kaiserslautern, Germany

**Keywords:** Brewers’ spent grain, bioprocess optimization, *Cellulomonas uda*, solid‑state fermentation

**Table of contents**

[**1.** **Materials & Methods** 2](#_Toc62047854)

[**1.1.** **Cell visualization** 2](#_Toc62047855)

[**1.2.** **HPLC analysis** 2](#_Toc62047856)

[**2.** **Results & Discussion** 3](#_Toc62047857)

[**2.1.** **2D fluorescence in aerobic and anaerobic fermentations** 3](#_Toc62047858)

[**2.2.** **Cell visualization** 4](#_Toc62047859)

[**2.3.** **Lignin‑like fluorescence** 5](#_Toc62047860)

[**2.4.** **Fermentation with pH shift** 5](#_Toc62047861)

[**3.** **List of chemicals** 5](#_Toc62047862)

# **Materials & Methods**

## **Cell visualization**

1 mL of culture broth, which was grown 4 days under aerobic or anaerobic conditions, was mixed with 3 µL of 5 mM green‑fluorescent nucleic acid stain (Syto 9, Invitrogen Corporation, Carlsbad, USA). After incubation in the dark for 15 min and centrifugation, the pellet was washed twice with 0.2 M MOPS buffer pH = 7.4. Fluorescence measurements were carried out on a confocal laser scanning microscope (SP5 II, Leica Microsystems GmbH, Wetzlar, Germany). Images were acquired using a 63 × 0.9 water immersion objective, with an argon laser at 15% AOTF@15% laser power, with a 488 nm excitation laser line and detection between 500 to 560 nm. Image size was 4096 px.

Cell images were taken with a light microscope Eclipse N*i* with a DS-I 1 camera equipped with a water immersion objective 40 × 0.65 ∞/0.17 WD 0.56 (all from Nikon Group, Tokyo, Japan).

## **HPLC analysis**

Organic acids were measured by high‑performance liquid chromatography (HPLC) with an Alliance 2695 (Waters Corporation, Milford, USA) and a reprogel H+ column, 300 mm x 4.6 mm, with integrated precolumn (Dr. Maisch GmbH, Ammerbuch, Germany) at 328 K, 9 mM H_2_SO_4_ and a flow of 0.3 mL∙min^-1^. The detection was performed with a 2996 photodiode detector (Waters corporation, Milford, USA) at a wavelength of 210 nm.

Sugar and ethanol were also measured by a modular HPLC system with a reprogel H+ column, 300 mm x 9 mm, with a security guard precolumn (Carbo‑H 4 mm x 3.0 mm ID, Phenomenex LTD, Torrance, USA) and 2.5 mM H_2_SO_4_ as eluent at a flow rate of 0.5 mL∙min^‑1^ and 298 K. The modular HPLC system consisted of a two‑channel degaser (Duratec GmbH, Hockenheim, Germany), a Merck Hitachi L‑6200 pump (Merck KGaA, Darmstadt, Germany), a Midas cool autosampler (Spark Holland B.V., Emmen, Netherlands) and a Jetstream II plus column thermostat (Duratec GmbH, Hockenheim, Germany). The detection was conducted with a refractive index detector (RI 101, Shodex, Kawasaki, Japan).

# **Results & Discussion**

## **2D fluorescence in aerobic and anaerobic fermentations**


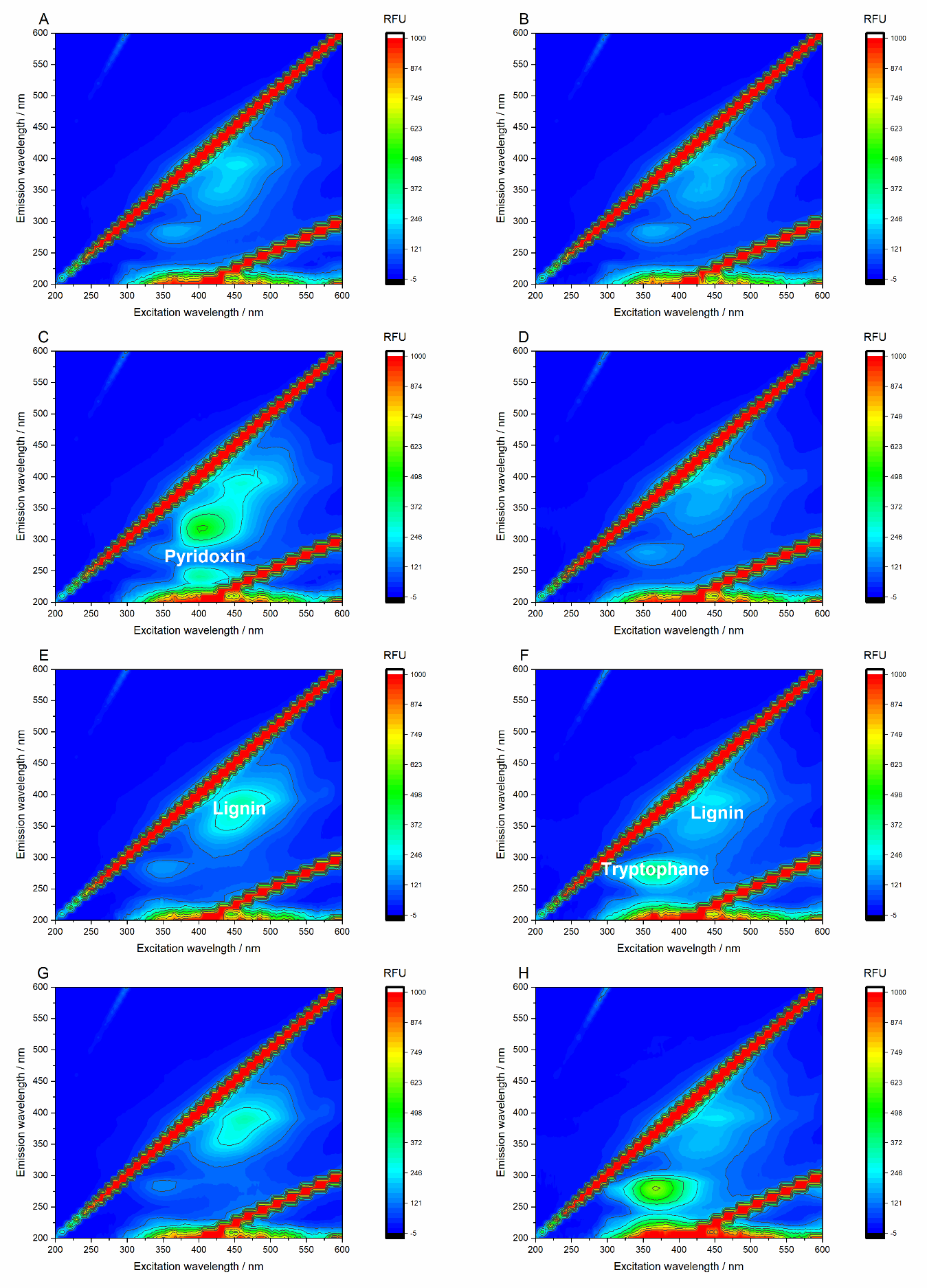


**Figure 1:** 2D fluorescence spectra of fermentations with C. uda in a medium consisting of 5w% ground BSG and 5 g∙L^-1^ yeast extract in 0.2 M MOPS buffer at pH = 7.4. Aerobic fermentation of day **A)** 0 **C)** 2 **E)** 4 **G)** 14 and anaerobic fermentation of day **B)** 0 **D)** 2 **F)** 4 **H)** 14; RFU, relative fluorescence units.

## **Cell visualization**


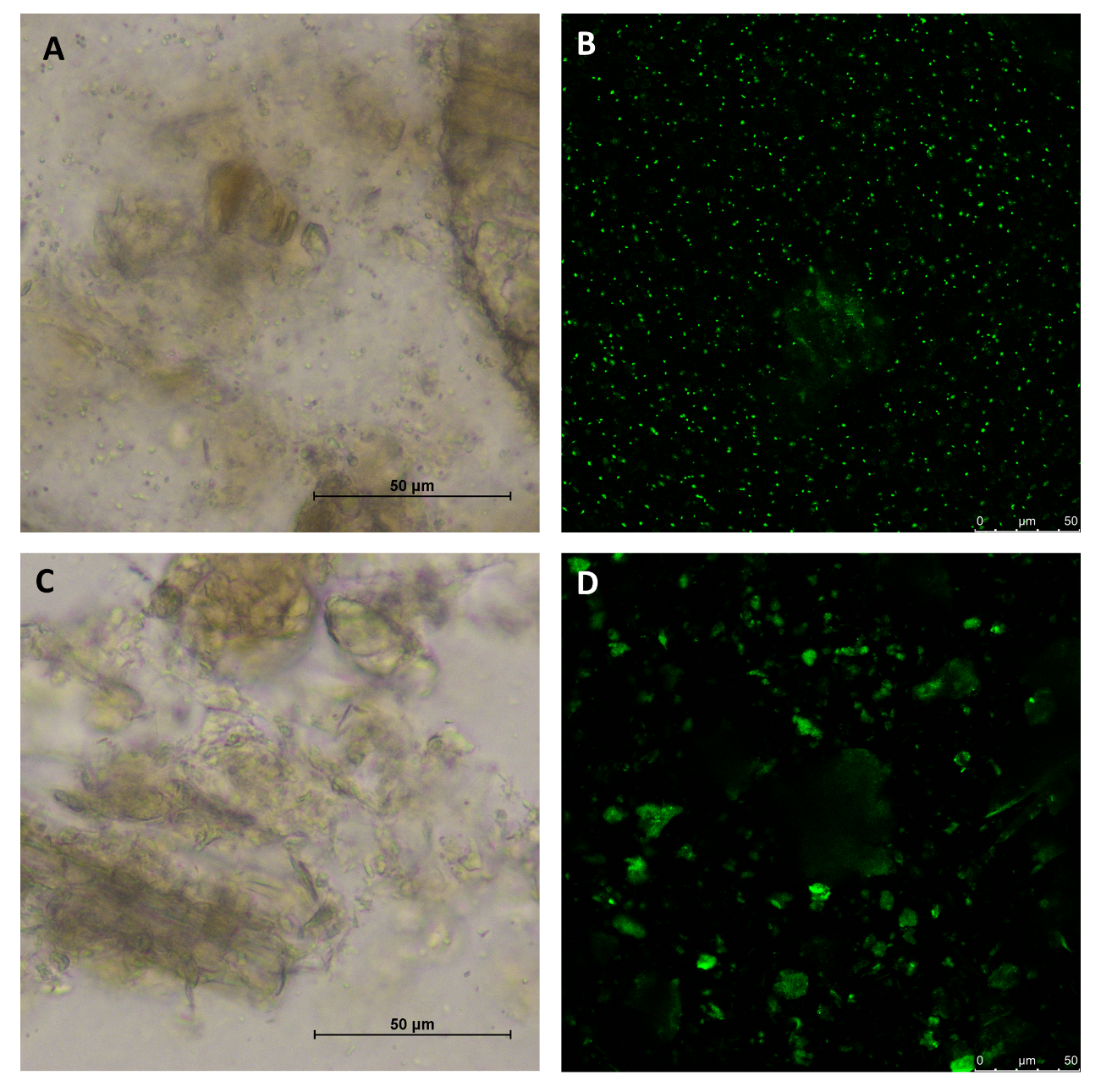


**Figure 2:** Visualization of C. uda after 4 days growth with 5w% ground BSG and 5 g∙L^-1^ yeast extract in 0.2 M MOPS buffer at pH = 7.4. **A)** light microscope, aerobic **B)** CLSM after staining, aerobic **C)** light microscope, anaerobic **D)** CLSM after staining, anaerobic; light microscopy images were cropped to square.

## **Lignin‑like fluorescence**





**Figure 3:** 2D fluorescence spectra of **A)** 10 g∙L^-1^ Ca^2+^-lignosulfonate in 0.2 M MOPS buffer at pH = 7.4 and **B)** undiluted fulvic acid; RFU, relative fluorescence units.

## **Fermentation with pH shift**





**Figure 4:** Fermentations with C. uda in a medium consisting of 5w% ground BSG and 5 g∙L^-1^ yeast extract in 0.2 M MOPS buffer. The pH was initially adjusted to pH = 7.4 and shifted to pH = 5 at day 4. **A)** Product concentrations of succinate (blue squares), lactate (red diamonds), formiate (turquoise triangles), acetate (pink circles) and ethanol (purple stars) during the fermentation **B)** released sugar due to bacterial degradation: cellobiose (medium grey triangles), xylose (dark grey squares) and arabinose (dark grey circles). The data show the mean values of 2 independent biological replicates.

# **List of chemicals**

The following chemicals were obtained from Carl Roth GmbH & Co. KG [CAS No.]: D(+)‑Glucose >99.5 % [50-99-7], L(+)-Arabinose [5328‑37‑0], D(+)-Xylose >99% [58‑86‑6], D(+)-cellobiose >98% [528‑50‑5], Yeast extract [8013-01-2], L‑Cysteine‑hydrochloride monohydrate >98.5 % [7048-04-6], NaOH >98 % [1310-73-2], Glycerol >99,5 % [56-81-5], Calcium lignosulfonate >93% [8061‑52‑7], Xylan from beechwood [232‑760‑6], MOPS buffer >99.5% [1132‑61‑2], acetic acid 100% [64‑19‑7], formic acid >98% [64‑18‑6], succinic acid >99% [110‑15‑6] and ethanol >99.8% [64‑17‑5]. Sigma‑Aldrich supplied: Potassium hexacyanoferrate >99% [13746‑66‑2], Pyridoxin hydrochloride >98% [58‑56‑0], L‑Tryptophan >98% [73‑22‑3], L(+)‑lactic acid 80‑92% [79‑33‑4], Sodium citrate tribasic dihydrate >99.5% [6132‑04‑3] and diethyl ether >99.5% [60‑29‑7]

Coomassie Bradford reagent [Art. No. 23236] was bought from ThermoFisher Scientific. Fulvic acid was provided by Dennerle GmbH.
